# A meta-analysis of total knee arthroplasty following high tibial osteotomy versus primary total knee arthroplasty

**DOI:** 10.1007/s00402-020-03333-6

**Published:** 2020-01-30

**Authors:** Xuedong Sun, Jun Wang, Zheng Su

**Affiliations:** 1 Department of Orthopaedics, Weifang People’s Hospital, No. 151 Guangwen Road, Weifang, 260041 China; 2 Department of Medical Oncology, Weifang People’s Hospital, No. 151 Guangwen Road, Weifang, 260041 China

**Keywords:** Knee osteoarthritis, High tibial osteotomy, Total knee arthroplasty, Meta-analysis

## Abstract

**Background:**

This study was performed to compare the clinical and radiographic outcomes of total knee arthroplasty (TKA) following high tibial osteotomy (HTO) versus primary TKA.

**Methods:**

Relevant trials were identified via a search of Ovid, PubMed and the Cochrane Central Register of Controlled Trials from inception to 10 January 2019. A meta-analysis was performed to compare postoperative outcomes between revising HTO to TKA (RHTO) and primary TKA (PTKA) with respect to Knee Society Score (KSS), 10-year survival rate, operative time, flexion and extension angle, infection rate and radiographic results.

**Results:**

Sixteen of 340 studies involving 103,552 adult patients (RHTO group, *n* = 3955; PTKA group, *n* = 99,597) were eligible for inclusion in the meta-analysis. Compared with primary TKA, revising HTO to TKA required longer operative time and had a higher infection rate (*P* < 0.05). The PTKA group had better flexion angle than the RHTO group (*P* < 0.05). There were no significant differences between the two groups in the KSS, extension angle, radiographic results and 10-year survival rate (*P* > 0.05).

**Conclusion:**

Patients who undergo conversion of HTO to TKA have similar 10-year survival rate, KSS, extension angle and radiographic results as patients who undergo primary TKA. However, conversion of HTO to TKA required longer operative time and had a higher infection rate than performing primary TKA. Moreover, conversion of HTO to TKA is associated with poorer flexion angle than primary TKA.

## Background

Total knee arthroplasty (TKA) and high tibial osteotomy (HTO) are both used to treat osteoarthritis of the knee. High tibial osteotomy is a well-established technique for the treatment of medial osteoarthritis of the knee with varus malalignment, especially in young and active patients [1, 2]. Some knees may need a conversion to TKA because of failure such as the progression of osteoarthritis. However, the outcome of TKA after HTO remains uncertain. Some authors [3, 4] reported that the results of TKA following HTO were similar to those of primary TKA, whereas others [5–7] described worse results and a higher number of complications in cases previously treated by tibial osteotomy. Therefore, we performed a meta-analysis of clinical studies to answer the following question: Does a previous HTO influence the function or survival of a TKA?

## Methods

### Search strategy

The Cochrane Central Register of Controlled Trials, Ovid and PubMed databases were searched to identify relevant studies published in English from inception to 10 January 2019. The following search strategy was used to maximize search specificity and sensitivity: [(revision hto) OR (revised hto) OR (revised high tibial osteotomy) OR (revision high tibial osteotomy)] AND [(total knee) OR tka OR tkr], where “tkr” stands for total knee replacement.

### Selection of studies

Three independent authors (X.D.S, J.W, and Z.S.) initially selected studies based on their titles and abstracts. Full papers were retrieved if a decision regarding study inclusion could not be made based on the titles and abstracts. The same three authors independently assessed each full paper to determine whether it met the inclusion criteria. Any disagreement was resolved by consensus; when a consensus could not be reached, the study was excluded.

### Inclusion criteria

Cohort studies, case control studies, and randomized controlled trials were eligible for inclusion if they met the following criteria:

- Comparison of the clinical outcomes of revised HTO versus primary TKA.
- Mean follow-up duration of at least 2 years.
- Evaluation of at least one of the following outcomes: Knee Society Score (KSS), 10-year survival rate, operative time, flexion and extension angles, infection rate, and radiographic results.
- Sufficient data for extraction and pooling (i.e., reporting of the mean, standard deviation, and number of subjects for continuous outcomes, and reporting of the number of subjects for dichotomous outcomes).

### Data extraction

Three reviewers (X.D.S. and J.W. and Z.S) independently performed data extraction using standardized data extraction forms. The general characteristics of each study were extracted [i.e., age, sex, body mass index (BMI), weight, follow-up, Knee Society Score (KSS), 10-year survival rate, operative time, flexion and extension angle, infection rate and radiographic results]. Any disagreement were resolved by consensus.

### Statistical analysis

Dichotomous outcomes are expressed as the risk ratio (RR) with 95% confidence interval (CI), while continuous outcomes are expressed as the mean difference (MD) or Standard mean difference (SMD) with 95% CI. Heterogeneity is expressed as *P* and *I*^2^. This value of *I*^2^ ranges from 0% (complete consistency) to 100% (complete inconsistency). If the *P* value of the heterogeneity test was < 0.1 or *I*^2^ > 50%, a random-effects model was used in place of the fixed modality. Publication bias was tested using funnel plots. Forest plots were used to graphically present the results of individual studies and the respective pooled estimate of effect size. All statistical analyses were performed with Review Manager (version 5.3.0 for Windows; Cochrane Collaboration, Nordic Cochrane Centre, Copenhagen, Denmark).

## Results

### Search results

**Fig. 1:**
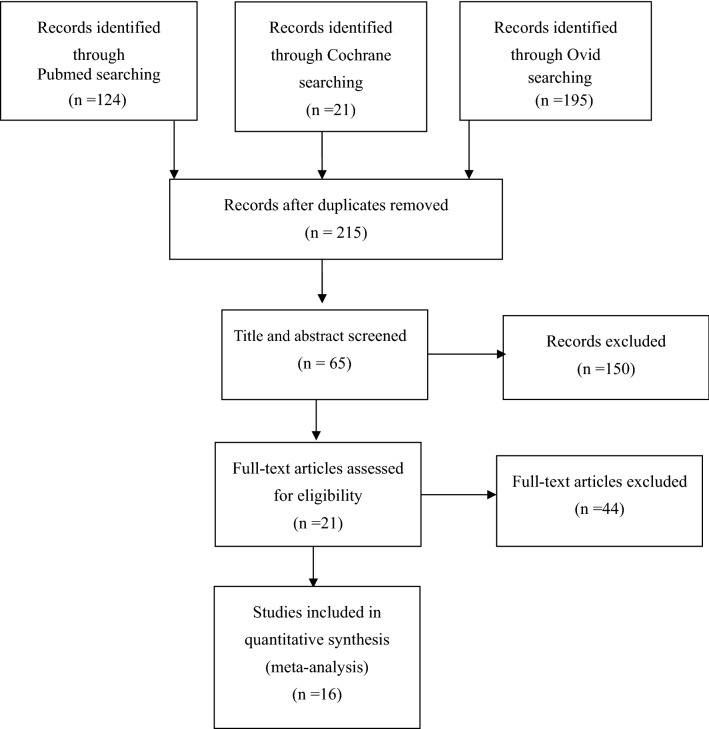
Flow of study selection

A flowchart of the studies considered for inclusion in our review is shown in Fig. 1. We identified 340 potential citations (124 from PubMed, 195 from Ovid, 21 from the Cochrane Library) comparing the clinical and radiographic outcomes of RHTO and PTKA. After reading the articles, Sixteen of the 340 citations were selected for the meta-analysis. The characteristics and data of these 16 studies [8–23] are shown in Tables 1 and 2.

**Table 1:** Characteristics of included studies

References	Technique of HTO	No. of patients	No. of knees	Age (years)	Female/male	BMI (kg/m^2^)	Mean time interval between HTO and TKA (months)	Follow-up (years)	Outcome
Amendola (2010) [8]	RHTO-C(19), RHTO-O(5)	24	29	68.5	19/5	NA	100.7	8.1	KSS, flexion angle, femorotibial angle
	PTKA	28	29	71	NA	NA	–	8.1	
Badawy (2015) [9]	RHTO	NA	1399	69	NA	NA	NA	> 10	Infections, 10-year survival rate
	PTKA	NA	31077	71	NA	NA	–	> 10	
Bae (2017) [10]	RHTO-C	29	32	68.3	29/0	26.6	150	6.2	KSS, flexion and extension angle, femorotibial angle, IS ratio
	PTKA	29	32	68.8	29/0	26.3	–	7.1	
Bergenudd (1997) [11]	RHTO	14	14	70	NA	NA	96	4–9	Flexion angle, infections, femorotibial angle, operative time
	PTKA	99	99	73	NA	NA	–	4–9	
Cross (2014) [12]	RHTO	43	43	54.2	12/32	33.36	106.2	8.47	KSS, infections, operative time
	PTKA	97	97	58.9	50/47	32.76	–	3.59	
Efe (2010) [13]	RHTO-C	41	41	69	20/21	NA	86	6.8	KSS, Infections, flexion and extension angle, IS ratio, operative time
	PTKA	41	41	73	24/17	NA	–	7.1	
El-Galaly (2018) [14]	RHTO	1044	1044	62	448/596	NA	NA	> 10	Infections, 10-year survival rate
	PTKA	63762	63762	70	41142/22621	NA	–	> 10	
Haddad (2000) [15]	RHTO-C(42), RHTO-D(8)	42	50	65	26/16	NA	87.6	6.2	KSS, femorotibial angle, infections
	PTKA	42	50	66	24/18	NA	–	6.2	
Haslam (2007) [16]	RHTO-C	40	51	78	20/19	NA	58	12.6	Infections
	PTKA	44	51	78	22/21	NA	–	12.6	
Karabatsos (2002) [17]	RHTO-C	20	17	64	10/10	NA	100.8	5.2	Infections, operative time
	PTKA	20	17	65	10/10	NA	–	4.7	
Kazakos (2008) [18]	RHTO-C	32	38	67.2	24/8	NA	87.6	4.5	KSS, flexion and extension angle, femorotibial angle, IS ratio, operative time
	PTKA	32	38	68.4	25/7	NA	–	4.5	
Meding (2011) [19]	RHTO-C	39	19	66.9	12/27	NA	104.4	16.7	KSS, flexion and extension angle, femorotibial angle
	PTKA	39	19	66.9	12/27	NA	–	16.6	
Niinimäki (2014) [20]	RHTO	NA	1036	64.3	NA	NA	NA	> 10	Infections, 10-year survival rate
	PTKA	NA	4143	64.7	NA	NA	–	> 10	
Nizard (1998) [21]	RHTO	55	57	71.8	NA	NA	116.4	4.5	KSS, IS ratio, infections
	PTKA	NA	57	70.5	NA	NA	–	4	
Saragaglia (2015) [22]	RHTO-O	40	45	69	10/30	29.7	NA	3.9	KSS, flexion angle, operative time
	PTKA	40	45	69	10/30	29	–	4.8	
Toksvig-Larsen (1998) [23]	RHTO	40	40	69	26/14	NA	120	10	Operative time
	PTKA	40	40	70	27/13	NA	–	10	

*No* number, *C* closing, *O* opening, *D* dome, *RHTO* revising high tibial osteotomy to total knee arthroplasty, *PTKA* primary total knee arthroplasty, *BMI* body mass index, *KSS* Knee Society Score, *IS ratio* Insall–Salvat ratio, *NA* not available

**Table 2:** The data of included studies

References		KKS	KFS	10-Survival rate	IS ratio	Femorotibial angle	Extension angle	Flexion angle	Operative time (min)	No of infections
Amendola (2010) [8]	RHTO	92.7	89.8	–	–	5.0°	–	103°	–	–
	PTKA	91	84	–	–	4.0°	–	100°	–	–
Badawy (2015) [9]	RHTO	–	–	92.6%	–	–	–	–	–	10/1399
	PTKA	–	–	93.8%	–	–	–	–	–	289/31077
Bae (2017) [10]	RHTO	90.6	88.8	–	1.13	6.0°	0.2°	129.2°	–	–
	PTKA	89.4	88.8	–	1.14	5.4°	0.2°	130.6°	–	–
Bergenudd (1997) [11]	RHTO	–	–	–	–	3°	–	95°	110	2/14
	PTKA	–	–	–	–	3°	–	95°	113	5/99
Cross (2014) [12]	RHTO	90	85	–	–	–	–	–	–	2/43
	PTKA	86	85	–	–	–	–	–	–	2/97
Efe (2010) [13]	RHTO	78.8	91	–	0.94	–	1.7°	106°	95	1/41
	PTKA	78.2	87.8	–	0.90	–	0.2°	115°	90	2/41
El-Galaly (2018) [14]	RHTO	–	–	91%	–	–	–	–	–	21/1044
	PTKA	–	–	94%	–	–	–	–	–	707/63762
Haddad (2000) [15]	RHTO	91	70	–	–	–	2°	95°	–	1/50
	PTKA	89	66	–	–	–	0°	103°	–	2/50
Haslam (2007) [16]	RHTO	–	–	–	–	–	–	–	–	3/51
	PTKA	–	–	–	–	–	–	–	–	0/51
Karabatsos (2002) [17]	RHTO	–	–	–	–	–	–	–	170	1/17
	PTKA	–	–	–	–	–	–	–	118	0/17
Kazakos (2008) [18]	RHTO	91.6	83	–	1.12	6.5°	0.56°	115.0°	75	–
	PTKA	92.0	84.4	–	1.24	5.4°	0.64°	119.1°	50	–
Meding (2011) [19]	RHTO	87.2	70.1	–	–	4.6°	− 0.1°	110.6°	–	–
	PTKA	90.3	72.6	–	–	4.6°	− 0.3°	112.6°	–	–
Niinimäki (2014) [20]	RHTO	–	–	91.8%	–	–	–	–	–	14/1036
	PTKA	–	–	94.5%	–	–	–	–	–	31/4143
Nizard (1998) [21]	RHTO	74.4	67.2	–	1.08	–	–	–	–	2/63
	PTKA	80.9	64.1	–	1.1	–	–	–	–	0/63
Saragaglia (2015) [22]	RHTO	87.5	96.5	–	–	–	–	117.5°	73.5	–
	PTKA	87.5	97	–	–	–	–	120.5°	68.5	–
Toksvig-Larsen (1998) [23]	RHTO	–	–	–	–	–	–	–	147	–
	PTKA	–	–	–	–	–	–	–	134	–

*No* number, *RHTO* revising high tibial osteotomy to total knee arthroplasty, *PTKA* primary total knee arthroplasty, *KKS* Knee Society Knee Score, *KFS* Knee Society Function Score, *IS ratio* Insall–Salvat ratio

### Meta-analysis results

**Fig. 2:**
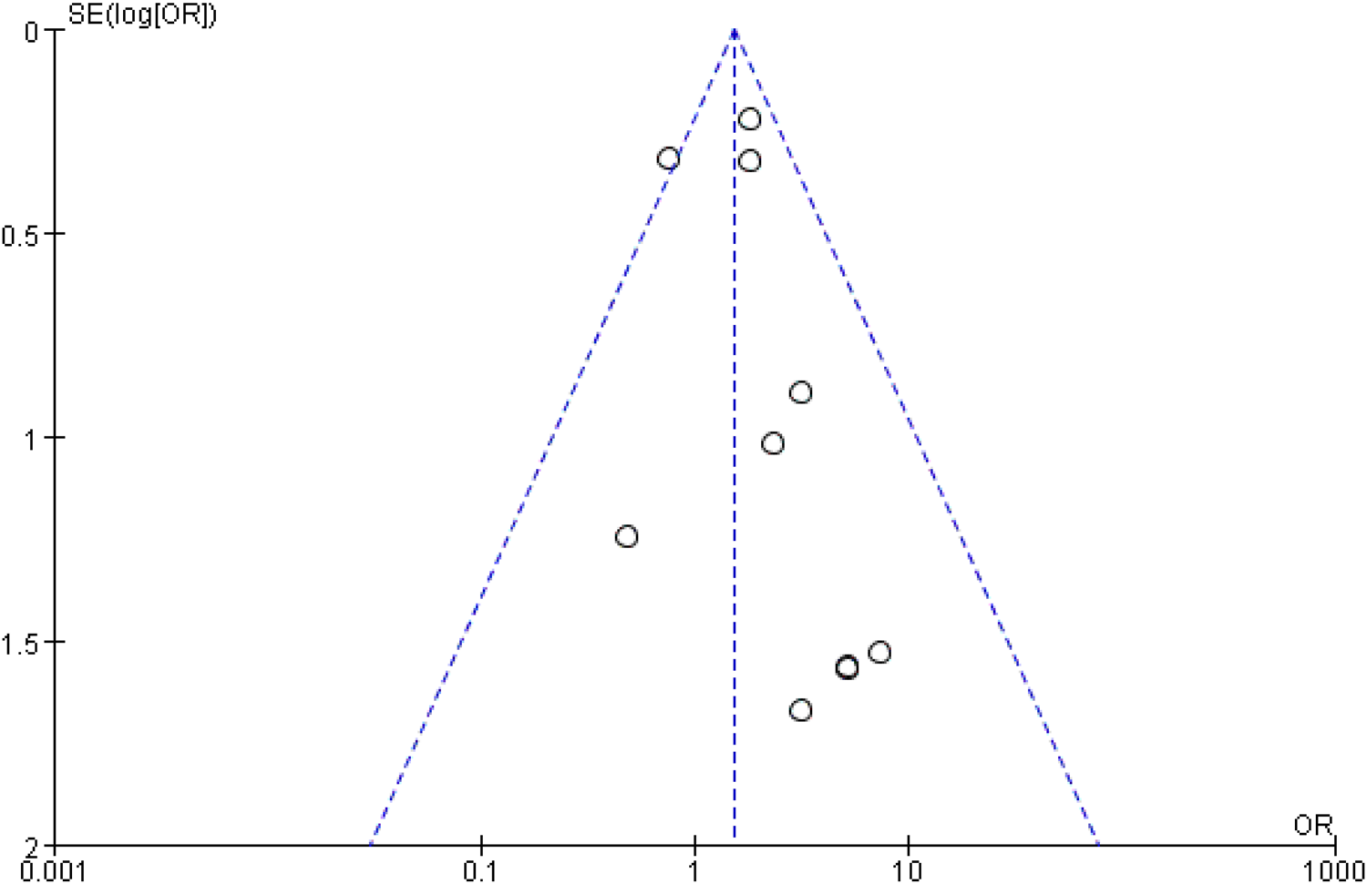
Funnel plot for infection rate

The meta-analysis included 16 studies [8–23], involving a total of 103,552 patients. The RHTO group included 3955 patients, while the PTKA group included 99,597 patients. A funnel plot based on the most frequently cited outcome was broadly symmetrical, indicating minimal publication bias (Fig. 2).

#### KSS

The KSS consists of the Knee Society Knee Score (KKS; 0–100) and the Knee Society Function Score (KFS; 0–100). There were no significant differences between these variables in the RHTO group versus the PTKA group (*P* > 0.05).

#### Radiographic results

**Fig. 3:**
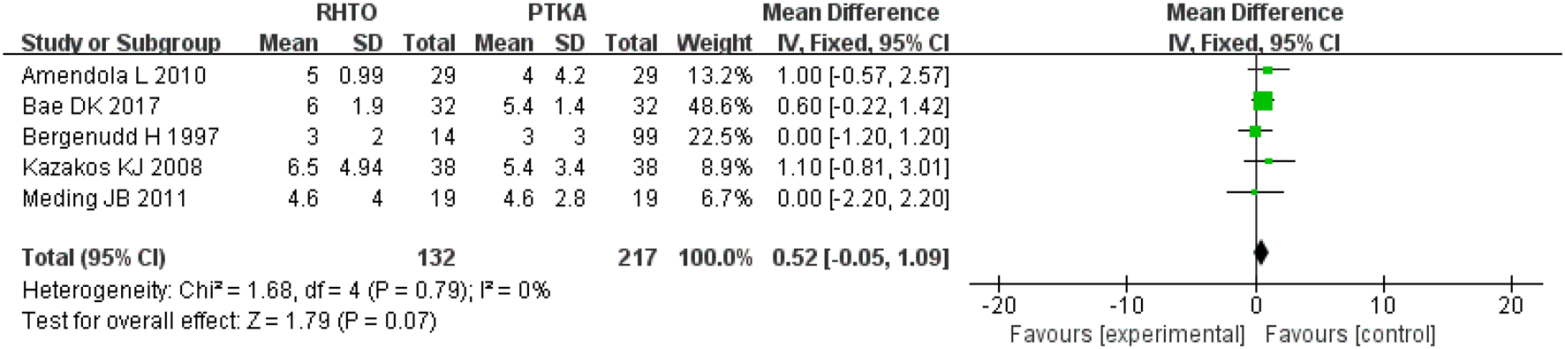
Forest plot for femorotibial angle

**Fig. 4:**
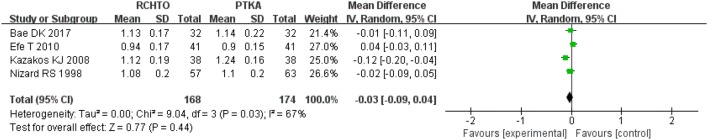
Forest plot for IS ratio

The radiographic results consist of femorotibial angle and IS ratio, and are summarized in Table 2. No significant differences were observed between the RHTO group and the PTKA group in terms of femorotibial angle and IS ratio (*P* > 0.05) (Figs. 3, 4).

#### Flexion and extension angle

The MD of the flexion and extension angle for TKA with HTO were − 2.92 (*P* = 0.006; 95% CI − 4.98 to − 0.86) and 0.66 (*P* = 0.11; 95% CI − 0.15 to 1.46), respectively. These results imply that flexion angle was better in the PTKA group than RHTO group, but the extension angle between the two groups was no significant differences.

#### Operative time and infection rate

Six studies involving 475 patients provided data on operative time. The operative time used for the RHTO group was significantly longer than that used for the PKA group (SMD = 1.10; 95% CI 0.20–2.00; *P* = 0.02).

Ten studies involving 103,158 patients provided data on infections. There was a significantly higher infection rate in the RHTO group than in the PTKA group (RR = 1.51; *P* = 0.005; 95% CI 1.14–2.02).

#### Survival rate

**Fig. 5:**
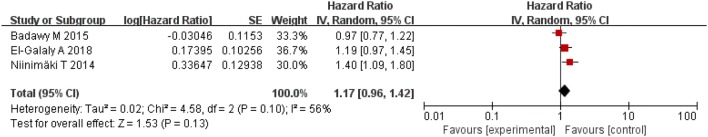
Forest plot for survival rate

Three studies involving 102,461 patients provided data on 10-year survival rate. No significant differences were found between the two groups (*P* > 0.05) (Fig. 5).

## Discussion

The most important finding of the present meta-analysis was that there were no significant differences between the RHTO group and the PTKA group regarding the KSS, extension angle, radiographic results, and 10-year survival rate. However, the PTKA group showed better outcomes than the RHTO group in terms of the flexion angle, operative time, and infection rate.

The KSS is often used to evaluate the ability to perform walking and stair-climbing activities.. Bae et al. [10] and Meding et al. [19] reported that the mean KSS did not significantly differ between the RHTO group and the PTKA group, whereas Efe et al. [13] found that the KSS was significantly better in the PTKA group than the RHTO group. The present meta-analysis revealed that the KSS was similar in the RHTO and the PTKA groups. The function of the knee is also assessed based on the range of motion (ROM) of the joint. In the present meta-analysis, the RHTO group had a smaller flexion angle than the PTKA group, which is in accordance with the findings of other studies [12, 13, 15]. Furthermore, extension angle did not significantly differ between the two groups. However, Miner et al. [24] revealed that ROM is much less important than the overall results, and Ripanti et al. [25] reported that previous HTO has no adverse effect on the outcome and functional results of the subsequent TKA.

HTO can result in a coronal deformity of the tibial plateau [26]. Lee et al. [27] found loss of correction in the operated limb occurred in open-wedge osteotomy, especially in the bilateral open-wedge osteotomy. Some studies [8, 11, 19] reported that the femorotibial angle after TKA following HTO did not significantly differ from that after primary TKA. Furthermore, some studies found that the patellar height was altered after HTO [6, 28]. Bae et al. [10] and Efe et al. [13] reported that the IS ratio did not significantly differ between the RHTO and PTKA groups; however, Kazakos et al. [18] reported the opposite. The present meta-analysis revealed that the mean femorotibial angle and IS ratio did not differ significantly between the two groups. The patellar height and femorotibial angle should be considered in preoperative planning. As the deformity can be corrected intra-articularly at the time of the TKA, it is essential to assess the postoperative alignment on radiography.

Previous studies have revealed that HTO results in satisfactory clinical outcomes. Furthermore, the HTO located at the metaphyseal areas had a significantly higher percentage of bone healing regardless of open- and closed-wedge osteotomies [29]. However, osteoarthritis progression may occur with a long term follow-up, and requiring conversion to TKA [30–32]. In addition, a systematic review and meta-analysis [33] found the HTO with concurrent cartilage procedures such as marrow stimulation procedure, mesenchymal stem cell transplantation, and injection were performed, but the concurrent procedures would produce little beneficial effect regarding clinical and radiological outcomes compared with HTO alone. In the included studies, the mean time interval between HTO and TKA ranged from 58 to 150 months (Table 1), which suggests that HTO is still a successful and reliable treatment method for unicompartmental knee osteoarthritis. However, previous studies have shown conflicting results regarding the survival rate after TKA following HTO versus that after primary TKA [34–36]. Several related studies with adequate sample sizes have been recently published, but the conclusions were still inconsistent. These studies estimated the survival rate using the Kaplan–Meier analysis and performed Cox regression analysis adjusted for age and sex. The present meta-analysis of the pooled 10-year survival rate data from the 102,461 patients evaluated in these included studies suggests that previous HTO should not be considered a factor related to a worse survival rate.

Conversion TKA after HTO may be more technically demanding than primary TKA due to the difficulty of the surgical approach, the ligamentous imbalance, and the anatomical distortion of the proximal tibial metaphysis [17, 37]. Bastos et al. [28] reported that incidence of additional procedures were required for the surgical approach because of the difficult patellar eversion in conversion TKA after HTO. Nagamine et al. [38] reported that a tibial offset stem may be required to solve the problem of translational and meta-diaphyseal mismatch of the tibia. Therefore, the longer operative time required for TKA following HTO is probably due to the increased surgical difficulty compared with primary TKA. Moreover, some studies [39, 40] reported that prolonged operative times were associated with an increased risk of surgical site infection, and related studies have also shown an increased risk of infection in patients undergoing TKA after prior knee surgery [41, 42]. The present meta-analysis revealed a higher infection rate in the RHTO group than in the PTKA group, which is in accordance with previous studies. This increased infection rate may be caused by the longer operative time and previous history of internal fixation.

The limitations of the present meta-analysis are the lack of adjustments for BMI or weight, the variation in the types of prostheses used, and the retrospective study design.

## Conclusion

Patients who undergo conversion of HTO to TKA have similar 10-year survival rate, KSS, extension angle and radiographic results as patients who undergo primary TKA. However, conversion of HTO to TKA required longer operative time and had a higher infection rate than performing primary TKA. Moreover, conversion of HTO to TKA is associated with poorer flexion angle than primary TKA.

## References

[1] Habata T, Uematsu K, Hattori K (2006). High tibial osteotomy that does not cause recurrence of varus deformity for medial gonarthrosis. Knee Surg Sports Traumatol Arthrosc.

[2] Lobenhoffer P (2014). Importance of osteotomy around to the knee for medial gonarthritis: indications, technique and results. Orthopade.

[3] Amendola A, Rorabeck CH, Bourne RB (1989). Total knee arthroplasty following high tibial osteotomy for osteoarthritis. J Arthroplasty.

[4] Staeheli JW, Cass JR, Morrey B (1987). Condylar total knee arthroplasty after failed proximal tibial osteotomy. J Bone Joint Surg Am.

[5] Katz MM, Hungerford DS, Krackow KA (1987). Results of knee arthroplasty after failed proximal tibial osteotomy for osteoarthritis. J Bone Joint Surg Am.

[6] Windsor RE, Insall JN, Vince KG (1988). Technical Considerations of total knee arthroplasty after proximal tibial osteotomy. J Bone Joint Surg Am.

[7] Tungall JA, Higgins GA, Waddell JP (2010). Complications of closing wedge high tibial osteotomy. Int Orthop.

[8] Amendola L, Fosco M, Cenni E, Tigani D (2010). Knee joint arthroplasty after tibial osteotomy. Int Orthop.

[9] Badawy M, Fenstad AM, Indrekvam K (2015). The risk of revision in total knee arthroplasty is not affected by previous high tibial osteotomy. Acta Orthop.

[10] Bae DK, Song SJ, Park CH (2017). Comparison of mid-term results between conversion total knee arthroplasties following closed wedge high tibial osteotomy and primary total knee arthroplasties: a matched pair study including patellar symptom and position. J Orthop Sci.

[11] Bergenudd H, Sahlström A, Sanzén L (1997). Total knee arthroplasty after failed proximal tibial valgus osteotomy. J Arthroplast.

[12] Cross MB, Yi PY, Moric M (2014). Revising an HTO or UKA to TKA: is it more like a primary TKA or a revision TKA?. J Arthroplast.

[13] Efe T, Heyse TJ, Boese C (2010). TKA following high tibial osteotomy versus primary TKA—a matched pair analysis. BMC Musculoskelet Disord.

[14] El-Galaly A, Nielsen PT, Jensen SL (2018). Prior high tibial osteotomy does not affect the survival of total knee arthroplasties: results from the Danish Knee Arthroplasty Registry. J Arthroplast.

[15] Haddad FS, Bentley G (2000). Total knee arthroplasty after high tibial osteotomy: a medium-term review. J Arthroplast.

[16] Haslam P, Armstrong M, Geutjens G (2007). Total knee arthroplasty after failed high tibial osteotomy long-term follow-up of matched groups. J Arthroplast.

[17] Karabatsos B, Mahomed NN, Maistrelli GL (2002). Functional outcome of total knee arthroplasty after high tibial osteotomy. Can J Surg.

[18] Kazakos KJ, Chatzipapas C, Verettas D (2008). Mid-term results of total knee arthroplasty after high tibial osteotomy. Arch Orthop Trauma Surg.

[19] Meding JB, Wing JT, Ritter MA (2011). Does high tibial osteotomy affect the success or survival of a total knee replacement?. Clin Orthop Relat Res.

[20] Niinimäki T, Eskelinen A, Ohtonen P (2014). Total knee arthroplasty after high tibial osteotomy: a registry-based case–control study of 1,036 knees. Arch Orthop Trauma Surg.

[21] Nizard RS, Cardinne L, Bizot P (1998). Total knee replacement after failed tibial osteotomy results of a matched-pair study. J Arthroplast.

[22] Saragaglia D, Massfelder J, Refaie R (2016). Computer-assisted total knee replacement after medial opening wedge high tibial osteotomy: medium-term results in a series of ninety cases. Int Orthop.

[23] Toksvig-Larsen S, Magyar G, Onsten I (1998). Fixation of the tibial component of total knee arthroplasty after high tibial osteotomy: a matched radiostereometric study. J Bone Joint Surg Br.

[24] Miner AL, Lingard EA, Wright EA (2003). Knee range of motion after total knee arthroplasty: how important is this as an outcome measure?. J Arthroplast.

[25] Ripanti S, Marin S, Romani G (2012). Total knee arthroplasty following high tibial osteotomy. J Bone Joint Surg Br.

[26] Farfalli LA, Farfalli GL, Aponte-Tinao LA (2012). Complications in total knee arthroplasty after high tibial osteotomy. Orthopedics.

[27] Lee OS, Kwon O, Lee YS (2018). Comparison of the outcome between unilateral and bilateral open wedge high tibial osteotomy in the bilateral varus knees. Arch Orthop Trauma Surg.

[28] Bastos Filho R, Magnussen RA, Duthon V (2013). Total knee arthroplasty after high tibial osteotomy: a comparison of opening and closing wedge osteotomy. Int Orthop.

[29] Simon MH, Grünwald L, Schenke M (2019). Corrective osteotomies of femur and tibia: which factors influence bone healing?. Arch Orthop Trauma Surg.

[30] Aglietti P, Buzzi R, Vena LM (2003). High tibial valgus osteotomy for medial gonarthrosis: a 10- to 21-year study. J Knee Surg.

[31] Coventry MB, Illstrup DM, Wallrichs SL (1993). Proximal tibial osteotomy. A critical long-term study of eighty cases. Bone Joint Surg Am.

[32] Yasuda K, Majima T, Tsuchida T (1992). A 10- to 15-year follow-up observation of high tibial osteotomy in medial compartment osteoarthrosis. Clin Orthop Relat Res.

[33] Lee OS, Ahn S, Ahn JH (2018). Effectiveness of concurrent procedures during high tibial osteotomy for medial compartment osteoarthritis: a systematic review and meta-analysis. Arch Orthop Trauma Surg.

[34] Pearse AJ, Hooper GJ, Rothwell AG (2012). Osteotomy and unicompartmental knee arthroplasty converted to total knee arthroplasty: data from the New Zealand joint registry. J Arthroplast.

[35] Parvizi J, Hanssen A, Spangehl MJ (2004). Total knee arthroplasty following proximal tibial osteotomy: risk factors for failure. J Bone Jt Surg Am.

[36] Treuter S, Schuh A, Hönle W (2012). Long-term results of total knee arthroplasty following high tibial osteotomy according to Wagner. Int Orthop.

[37] Bae DK, Song SJ, Yoon KH (2010). Total knee arthroplasty following closed wedge high tibial osteotomy. Int Orthop.

[38] Nagamine R, Inoue S, Miura H (2007). Femoral shaft bowing influences the correction angle for high tibial osteotomy. J Orthop Sci.

[39] Pugely AJ, Martin CT, Gao Y (2015). The incidence of and risk factors for 30-day surgical site infections following primary and revision total joint arthroplasty. J Arthroplast.

[40] George J, Mahmood B, Sultan AA (2018). How fast should a total knee arthroplasty be performed? An analysis of 140,199 surgeries. J Arthroplast.

[41] Berbari EF, Hanssen AD, Duffy MC (1998). Risk factors for prosthetic joint infection: case–control study. Clin Infect Dis.

[42] Oiestad BE, Engebretsen L, Storheim K (2009). Knee osteoarthritis after anterior cruciate ligament injury: a systematic review. Am J Sports Med.

